# Foot Strike Patterns During Overground Distance Running: A Systematic Review and Meta-Analysis

**DOI:** 10.1186/s40798-021-00369-9

**Published:** 2021-11-10

**Authors:** Stephen P. Bovalino, Michael I. C. Kingsley

**Affiliations:** 1 Holsworth Research Initiative, La Trobe Rural Health School, La Trobe University, Melbourne, VIC Australia; 2 Exercise Sciences, Faculty of Science, University of Auckland, Auckland, New Zealand

## Abstract

**Background:**

Investigations of foot strike patterns during overground distance running have foci on prevalence, performance and change in foot strike pattern with increased distance. To date, synthesised analyses of these findings are scarce.

**Objective:**

The key objectives of this review were to quantify the prevalence of foot strike patterns, assess the impact of increased running distance on foot strike pattern change and investigate the potential impact of foot strike pattern on performance.

**Methods:**

Relevant peer-reviewed literature was obtained by searching EBSCOhost CINAHL, Ovid Medline, EMBASE and SPORTDiscus (inception-2021) for studies investigating foot strike patterns in overground distance running settings (> 10 km). Random effects meta-analyses of prevalence data were performed where possible.

**Results:**

The initial search identified 2210 unique articles. After removal of duplicates and excluded articles, 12 articles were included in the review. Meta-analysis of prevalence data revealed that 79% of long-distance overground runners rearfoot strike early, with prevalence rising to 86% with increased distance. In total, 11% of runners changed foot strike pattern with increased distance and of those, the vast majority (84%) do so in one direction, being non-rearfoot strike to rearfoot strike. Analysis of the relationship between foot strike pattern and performance revealed that 5 studies reported a performance benefit to non-rearfoot strike, 1 study reported a performance benefit to non-rearfoot strike in women but not men, 4 studies reported no benefit to non-rearfoot strike or rearfoot strike, and no studies reported a performance benefit of rearfoot strike over non-rearfoot strike.

**Conclusion:**

Most overground distance runners rearfoot strike early, and the prevalence of this pattern increases with distance. Of those that do change foot strike pattern, the majority transition from non-rearfoot to rearfoot. The current literature provides inconclusive evidence of a competitive advantage being associated with long-distance runners who use a non-rearfoot strike pattern in favour of a rearfoot strike pattern.

**Supplementary Information:**

The online version contains supplementary material available at 10.1186/s40798-021-00369-9.

## Key Points

- In total, 79% of overground distance runners rearfoot strike early, with prevalence increasing to 86% as distance increases.
- In total, 11% of overground distance runners change their foot strike pattern as distance increases, with the majority of them transitioning from a non-rearfoot strike pattern to a rearfoot strike pattern
- The evidence in support of a non-rearfoot strike pattern conferring a competitive performance advantage over the rearfoot strike pattern is inconclusive.

## Introduction

Foot strike patterns in runners are generally grouped into three categories: rearfoot strike (RFS), midfoot strike (MFS) and forefoot strike (FFS). Classification of runners into one of these three categories can be achieved by observing the first point of contact between the landing foot with its running surface. The point of initial contact can be categorised to have occurred in one of three anatomical loci, which serve to describe the overall observed pattern. Broadly, an RFS pattern is said to occur when initial contact is made on the heel or rear one-third of the foot, MFS when the heel and ball of the foot contact nearly simultaneously, and FFS when first contact is made on the front half of the foot, after which heel contact follows [1–3]. Due to the relatively low prevalence rates of both MFS and FFS patterns, coupled with the fact that they both occur at the anterior aspect of the foot, a further sub-classification exists which combines the two. This combined category is sometimes referred to as an anterior foot strike pattern, but more commonly has been described in the literature as a non-rearfoot strike (NRFS) pattern [1].

Foot strike patterns and their relationships with running performance, injury and economy have served as topics for debate within the literature, with some authors suggesting that changing foot strike is not beneficial to runners [4]. Claims of improved running economy [5] and reduced rates of injury [6] have been reported in those habitually using NRFS patterns compared to those employing RFS; however, it is important to note that these associations are equivocal and the potential benefit of using an NRFS pattern has been challenged in the literature [4, 7].

The tightly controlled nature of the laboratory environment confers a number of obvious key advantages when investigating foot strike patterns. The use of a treadmill allows for static analysis in multiple planes, with precise adjustment of speed as a variable easily administered. Many studies in the past have employed this technique when investigating foot strike patterns [8–10]. However, when compared to overground running, treadmill running has been shown to alter key elements of the kinematic gait cycle; differences have been reported in the angle between shoe sole and ground at foot contact as well as step length, stride frequency and foot contact time [11]. When comparing novice and competitive runners, untrained individuals are more prone to this phenomenon, with novice runners showing larger kinematic adjustments in a fatigued state when compared to their competitive counterparts [12]. It follows that research pertaining to foot strike pattern analyses performed in a laboratory or using a treadmill might not be applicable outside these settings. Focusing analyses on overground running specifically omits this potential confounding and confers wide applicability to the significant cohort of runners who engage in overground distance running.

Foot strike patterns during overground distance running (> 10 km) have become increasingly researched. Other than the preliminary work by Kerr et al. [13], the paper by Hasegawa et al. was the first well-designed and executed attempt to quantify and analyse foot strike patterns in an overground distance running setting, where capture occurred at the 15 km distance of an elite half marathon event [2]. This analysis by Hasegawa et al. was the first to be conducted within the confines of an official long-distance running event, with access to large numbers of participants subjected to the same race distance and environment providing optimal conditions for investigation. Since this work there have been additional attempts to explore foot strike patterns during overground running, using similar methods. Subanalyses on the relationship between foot strike patterns and performance [14, 15], as well as assessment of the role that fatigue plays on foot strike patterns [3], are also offered within this setting, as access to published race results is often freely available and matching participants over multiple time points in an event is possible.

Multiple studies have been published that investigate foot strike patterns within the context of long-distance overground running. To date, no systematic review and meta-analysis has been published to collate and quantify this literature base. Through establishing key prevalence data, observing the impact of distance and assessing any potential performance benefit associated with foot strike patterns, runners and coaches are permitted access to a foundation of knowledge to which training applications can be based on. The aims of this systematic review were to: (1) establish the prevalence of RFS and NRFS patterns both early and late in overground distance running settings; (2) assess the impact of increased distance on foot strike pattern change and establish its direction; and (3) determine whether the NRFS pattern confers a performance advantage over the RFS pattern in long-distance overground running; defined as either a faster finishing time or better representation in finishing position.

## Methods

This review was reported in accordance with the Preferred Reporting Items for Systematic Reviews and Meta-Analyses (PRISMA) statement [16] (Additional file 1: Table S1).

### Search Strategy

Articles from the literature were systematically identified by searching the following databases from inception to the 2 July 2021; EBSCOhost CINAHL, Ovid Medline, EMBASE and SPORTDiscus. The search strategy was designed using terms within the three major constructs related to the research question (runners, distance setting and foot strike patterns), combined with the AND operator. These three constructs were chosen to ensure results were focused on populations of runners engaging in a distance sub-discipline where foot strike patterns were analysed. Similar key terms were entered, in parenthesis, and separated by the term OR and truncation was used (*) to capture all possible variations of the selected key terms. The following search strategy was used: (running (MeSH) OR jogging (MeSH) OR runner* OR jogger* OR run OR jog) AND (distance OR length OR “long distance” OR marathon OR “half marathon” OR “ultra marathon” OR “race”) AND (“foot strike” OR forefoot OR midfoot OR rearfoot OR “ground contact” OR “foot contact” OR footfall OR “foot landing”). In addition to the database search, the reference lists of relevant articles were also reviewed. No filters were employed in the search. The literature search was undertaken by author SB.

### Inclusion and Exclusion Criteria

Original cross-sectional cohort studies published in English from peer-reviewed journals between no date to 2 July 2021 that focused primarily on capturing foot strike patterns in distance overground running settings were included. Articles were excluded based on the following criteria: (1) study conducted in a laboratory or on a treadmill, (2) < 10 km total run distance, (3) not available in English language, (4) not peer-reviewed original research, (5) foot strike patterns not observed, (6) study conducted on non-human animals, (7) foot strike captured before 2 km or within 1 km of the finish (so as to combat potential surges in speed which can influence foot strike pattern), (8) conference proceedings, (9) study conducted on a non-random sample of participants and (10) non-observational study (intervention administered). Title review was undertaken by 1 reviewer (SB), followed by independent review of the abstract and full text articles by 2 reviewers (SB & MK) using the pre-agreed inclusion and exclusion criteria (Cohen’s Kappa = 0.823). Disagreements were resolved after discussion between the 2 reviewers.

### Outcomes of Interest

In line with the research question and search strategy, data relating to three main areas of interest were collected and reported on: (1) foot strike pattern prevalence (including asymmetry); (2) the influence of increased distance on individual matched foot strike patterns, which was defined as change from NRFS to RFS or the converse between the first and last checkpoints; and (3) the relationship between foot strike pattern and performance. One author (SB) performed the data extraction and the other author (MK) confirmed accuracy of the extracted data with no disagreements encountered. Separate studies employed different methods to quantify the impact of foot strike pattern on performance (i.e. finishing time, finishing position or representation within specific finishing centiles). To combine these data, a binary transformation was applied to the performance results of each study as either NRFS being faster than RFS, RFS being faster than NRFS or no difference.

### Critical Review of Study Quality

A critical analysis of the included literature was undertaken to determine study quality. Given all included articles were observational cross-sectional studies, an adapted version of the NIH Quality Assessment Tool for Observational and Cross-Sectional Studies was employed. The original tool allocated a maximum of 14 points for the highest quality study; it was established that Sects. 6, 13 and 14 of the original tool were not applicable to our particular cohort of studies, and thus, our adapted tool allocated a maximum of 11 points for the highest quality studies. To score a point for question 2 relating to study population, standard of athlete and or event name had to be specified. A point was given for question 8 (exposure) when studies analysed running speed, sampled from bands of running speed or commented on markers of intensity/effort. All remaining points were given in accordance with the originally designed tool. It was determined that studies for this review that scored between 9 and 11 were of high quality, 7–8 moderate quality and < 7 low quality. Quality assessment was performed by both authors SB and MK, and disagreements were resolved by discussion.

### Meta-Analysis of Prevalence Data

Meta-analyses of prevalence data were generated using the software package MetaXL (Version 5.3; EpiGear International Pty Ltd, Australia) employing a random effects model with double arcsine transformation [17]. The proportion of effects due to heterogeneity was assessed using the *I*^2^ statistic, where low, moderate and high levels of heterogeneity were determined by *I*^2^ values of < 25%, 25–75% and > 75%, respectively [18].

## Results

### Search Strategy

**Fig. 1:**
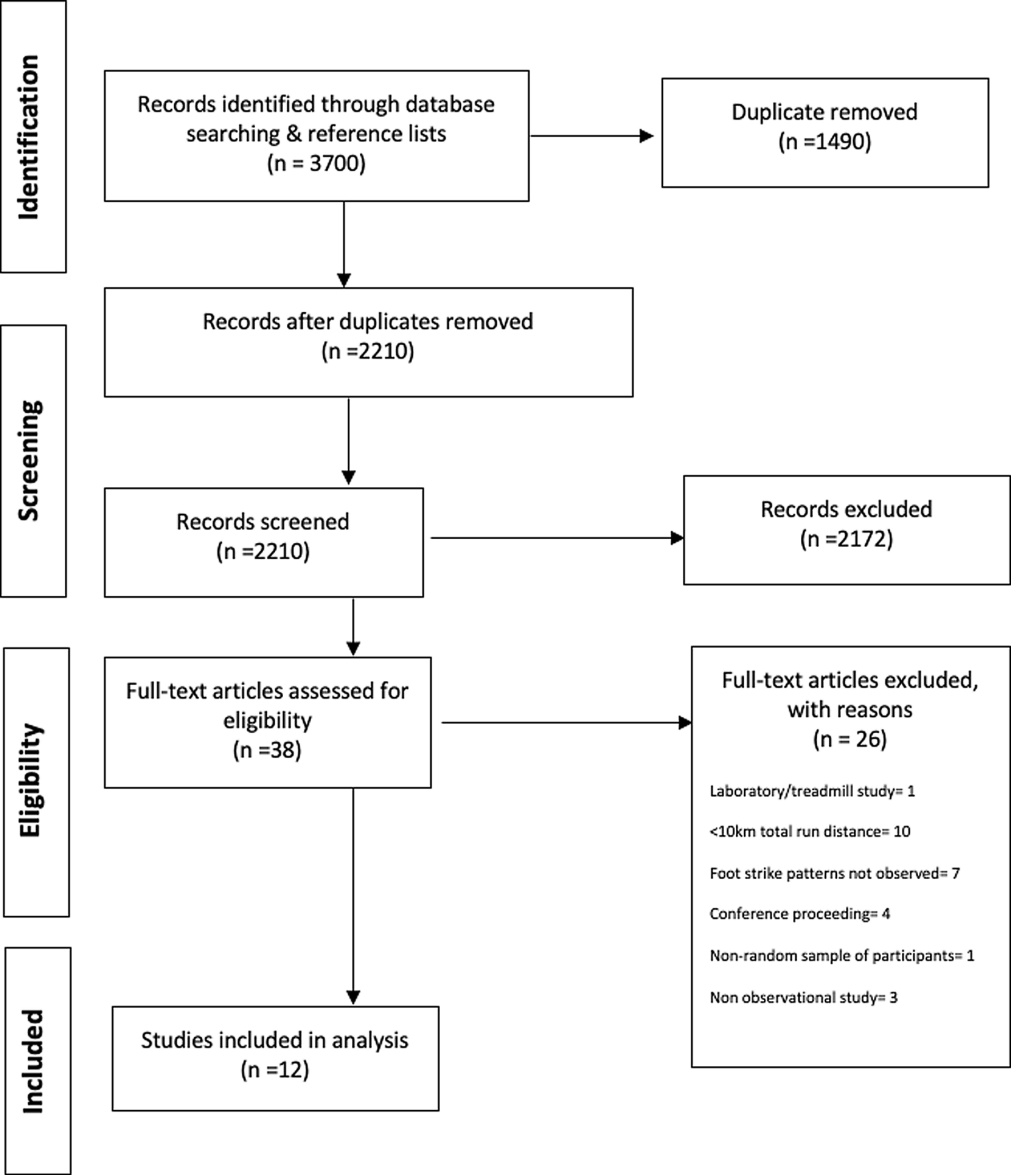
PRISMA flow diagram

A total of 2210 unique articles were identified during the initial search strategy and through searching of reference lists. Of these, 12 articles met the inclusion criteria and were included in the analyses (Fig. 1). Table 1 summarises the study characteristics, research design, total race distance, checkpoint/foot strike prevalence data, performance analysis and effect of increased distance on foot strike patterns assessing for change.

**Table 1:** Analysis of included studies

Study: country	Sample (total *N*, location, athlete level)	Study Design	Distance (total)	Distance (checkpoint) (km)	RFS (%)	NRFS (%)	MFS (%)	FFS (%)	Asymmetrical	Performance (NRFS vs RFS)	Change in foot strike pattern between first and last checkpoints (NRFS to RFS or RFS to NRFS)^c^
Bovalino et al. [1]: Australia	*N* = 459 participants at the 2017 Melbourne City to Sea recreational running event (recreational)	Cross sectional	15 km	3	76.9	22.0	N/A	N/A	N/A	NRFS faster than RFS	*N* = 67/459 (14.6%) changed foot strike *N* = 64/67 (95.5%) changed from NRFS to RFS *N* = 3/67 (4.5%) changed from RFS to NRFS
				13	91.0	8.7	N/A	N/A	N/A		
Hanley et al. [19]: UK^d^	*N* = 12 participants at the 2017 IAAF World Championships men’s 10,000 m final (elite)	Cross sectional	10 km	4.18	0	100	42	50	8%	N/A^d^	*N* = 0/12 changed foot strike (0%)^d^
				5.78	0	100	50	42	8%		
				7.78	0	100	50	33	17%		
Hebert-Losier et al. [21]: Singapore	*N* = 350 participants at the 2015 Standard Chartered Singapore Marathon (recreational)	Cross sectional	42.2 km	10	65	24	21	3	11%	NRFS NOT faster than RFS	N/A
				39	77	16	15	1	8%		
Hanley et al. [14]: UK	*N* = 149 participants at the 2017 IAAF World Championships marathon event (elite)	Cross sectional	42.2 km	8.5	60	40	36	4	N/A	NRFS NOT faster than RFS for men NRFS faster than RFS for women	*N* = 30/149 (20.1%) changed foot strike *N* = 24/30 (80%) changed from NRFS to RFS *N* = 6/30 (20%) changed from RFS to NRFS
				19	64	35	31	4	N/A		
				29.5	65	35	32	3	N/A		
				40	70	30	27	3	N/A		
Hasegawa et al. [2]: Japan	*N* = 283 participants at the 2004 47th Sapporo International Half Marathon (elite)	Cross Sectional	21.1 km	15	74.9	25.1	23.7	1.4	N/A	NRFS faster than RFS	N/A
Kasmer et al. [22]: USA	*N* = 161 participants at the 2012 Ice Age Trail 50 km race (recreational)	Cross sectional	50 km	8.1	85.1	13.7	13.7	0	1.2%	NRFS NOT faster than RFS	N/A
Kasmer et al.[20]: USA	*N* = 1,991 participants at the 2011 Milwaukee Lakefront Marathon (recreational)	Cross sectional	42.2 km	8.1	93.7	5.6	5.1	0.6	0.7%	NRFS faster than RFS	N/A
Kasmer et al. [23]: USA^a^	*N* = 316 participants at the 161.1 km Western States Endurance Run (recreational)	Cross sectional	161.1 km	16.5	79.9	11.1	7.8	0.3	N/A	NRFS NOT faster than RFS	*N* = 23/316 (7.3%) changed foot strike pattern *N* = 17/23 (73.9%) changed from NRFS to RFS *N* = 6/23 (26.1%)) changed from RFS to NRFS
				90.3	89	6.8	3.2	1.1	N/A		
Latorre-Roman et al. [24] Spain^e^	*N* = 542 athletes who participated in the 2011 XVII International Half Marathon of Cordoba (recreational)	Cross sectional	21.1 km	15	95.4	4.6	3.5	1.1	25.9%	NRFS faster than RFS	N/A
Larson et al. [3]: USA^b^	*N* = 286 participants in the 2009 Manchester City Marathon (recreational)	Cross sectional	42.2 km	10	87.8	4.5	3.1	1.4	7.7%	NRFS NOT faster than RFS	*N* = 30/286 (10.5%) changed foot strike pattern *N* = 23/30 (76.7%) changed from NRFS to RFS *N* = 7/30 (23.3%) changed foot strike from RFS to NRFS
				32	93	3.5	3.5	0	3.5%		
	*N* = 650 participants of the marathon relay and half marathon 2009 Manchester City Marathon (recreational)	Cross sectional	21.1 km marathon relay and half marathon cohort	10	89.4	5.5	3.5	2	5.1%	NRFS NOT faster than RFS	N/A
Murray et al. [25]: New Zealand	*N* = 24 recreationally competitive runners (recreational)	Cross sectional	12 km	3	96	4	4	0	N/A	N/A	*N* = 1/24 (4%) changed foot strike pattern *N* = 1/1 (100%) changed from NRFS to RFS *N* = 0/1 (0%) changed from RFS to NRFS
				10	100	0	0	0	N/A		
Patoz et al. [26]: Singapore	*N* = 940 participants of the 2015 Standard Chartered Singapore Marathon (recreational)	Cross sectional	42.2 km	10	71.1	18.3	16.6	1.7	10.6%	NRFS faster than RFS	N/A

*FFS* forefoot strike, *MFS* midfoot strike, *NRFS* non-rearfoot strike, *N* number of subjects, *RFS* rearfoot strike

^a^Kasmer et al. [23] four checkpoints included in study, but final checkpoint removed as it violated exclusion criteria of being within 1 km of finish line. Checkpoint at 90.7 km removed as it was downhill and deemed likely to impact foot landing position. Values do not add up to 100% due to different methods of foot strike categorisation used which could not always be adapted to fit table

^b^Larson et al. [3] Relay marathon and half marathon cohort table has been adapted from raw data within study by subtracting the pure marathon runner cohort 10 km data (*n* = 286) from the combined marathon/relay and half marathon 10 km data (*n* = 936) to create a novel dataset of *N* = 650 10 km data for relay marathon and half marathon combined. “Change” foot strike data were adapted from the raw data set provided by original author which contained both left and right individual foot strike data between checkpoints. These data were re-categorised to accept change between checkpoints in either left or right foot, to be deemed as change in general to ensure data were congruent with other studies. In cases where different foot strike patterns were observed between feet, but this pattern did not change between checkpoints, a NO change categorisation was given

^c^% and *N* = values will not always correlate with values offered in publication due to constraints on available data. Relevant text or tables within the paper used to produce congruent and applicable values for the purposes of this review. Direction of foot strike pattern change only recorded as either NRFS to RFS or RFS to NRFS. Data not included if studies reported further sub-categorisation of FFS or MFS

^d^Hanley et al. [19] = 6 checkpoints included in study, first two checkpoints removed due to violating exclusion criteria of being within 2 km of start line and final checkpoint removed as it violated exclusion criteria of being within 1 km of finish line. Performance data considered to be N/A as no competitors recorded as RFS for comparison. Under the definition given of changed foot strike contained within the methods of this study, no participants were seen to change from NRFS to RFS (please note original paper did observe sub-category change between FFS and MFS)

^e^Latorre-Roman et al. [24] values were re-categorised from individual foot strike patterns. Asymmetry value % not offered in context of other foot strike patterns in paper

### Study Design

Studies differed with regard to country of implementation, study design (sampling, recruitment, measurement methods) and primary outcomes (foot strike pattern, performance and change in foot strike pattern). Four studies were conducted in the USA, 2 in Singapore, 2 in the UK and with 1 study being conducted in each of Australia, Japan, New Zealand and Spain (Table 1). All studies were cross-sectional cohort in their design. Sample sizes ranged from 12 participants [19] to 1991 [20]. Three studies were performed on entirely elite cohorts [2, 14, 19], while the remaining studies were all performed on recreational cohorts [1, 3, 20–26]. Total run distance varied from 10 km [19] to a 161.1 km ultramarathon [23]. Five studies were performed within the confines of a traditional marathon distance (42.2 km) [3, 14, 20, 21, 26] and 2 at half marathon distance (21.1 km) [2, 24]. The remaining studies were conducted over a 12-km track run [25], 15-km road run [1] and a 50-km trail run [22]. All studies had at least one capture checkpoint for foot strike pattern analysis, with 4 studies employing two checkpoints [1, 3, 21, 25] and 3 studies with more than two checkpoints [14, 19, 23]. Studies that included 2 or more checkpoints enabled for assessment of the relationship between increased distance and change in foot strike pattern. Distances at which the various checkpoints occurred varied between studies (Table 1). All studies assessed for RFS, MFS and FFS prevalence at each checkpoint except for 1 study, which assessed for RFS and NRFS (combination of either FFS or MFS) [1]. Seven studies assessed prevalence of asymmetrical foot strike patterns (difference between left and right foot strike) [3, 19–22, 24, 26]. All studies assessed for impact of foot strike pattern on performance except 1 study [25].

### Foot Strike Pattern Prevalence

**Fig. 2:**
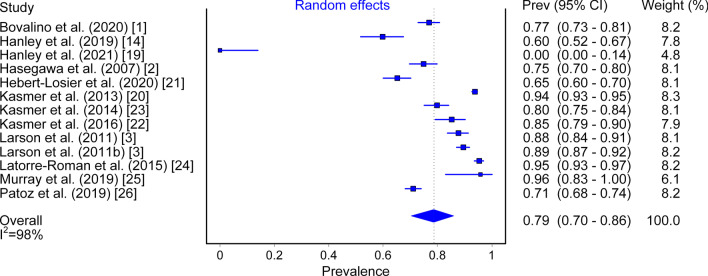
Prevalence of RFS measured at the first (or only) checkpoint

**Fig. 3:**
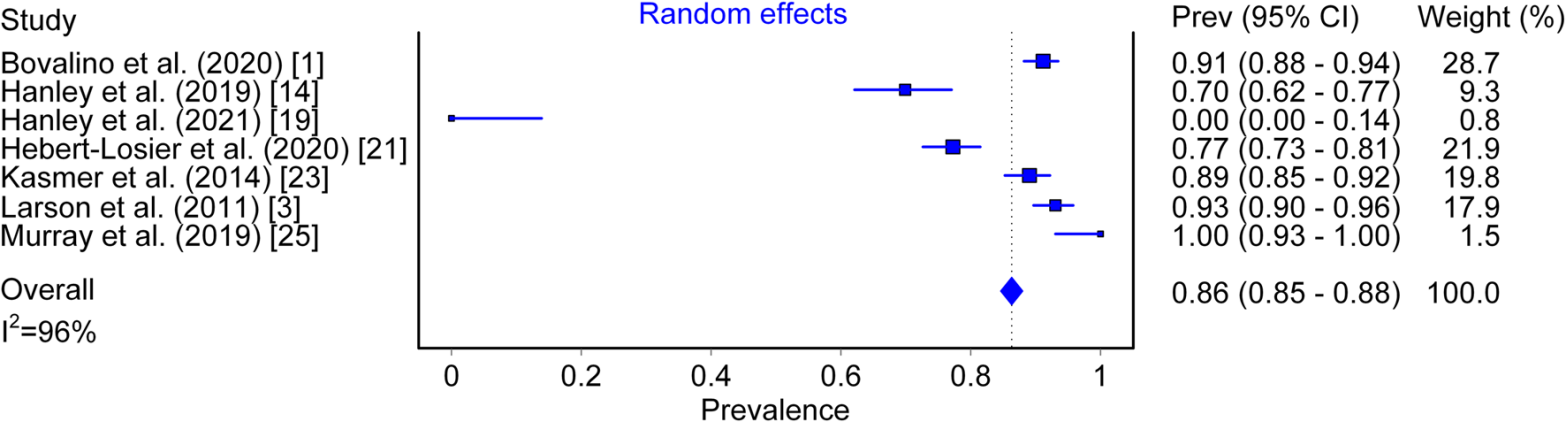
Prevalence of RFS measured at the final checkpoint in studies that used > 1 checkpoint

Overall RFS prevalence at the first (or only) checkpoint was 79% (95% CI 0.70–0.86, *I*^2^ = 98%; Fig. 2), while overall prevalence for the RFS pattern at the final checkpoint (in studies that included more than one checkpoint) was 86% (95% CI 0.85–0.88, *I*^2^ = 96%; Fig. 3).

### Foot Strike Pattern Change

**Fig. 4:**
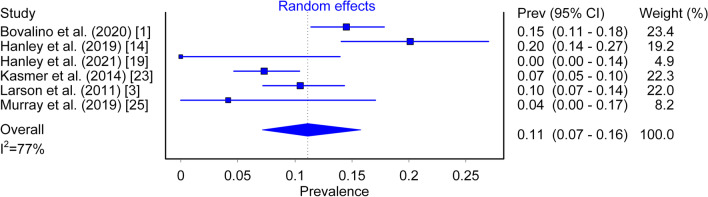
Prevalence of total change between first and last checkpoints

**Fig. 5:**
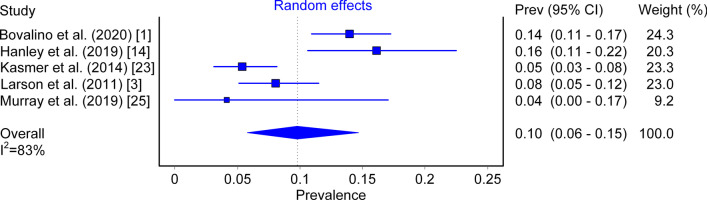
Prevalence of change from NRFS to RFS between first and last checkpoints

Prevalence of total change in foot strike pattern was observed to be 11% (95% CI 0.07–0.16, *I*^2^ = 77%; Fig. 4) and of this specific cohort the proportion seen to change from NRFS to RFS was 84% (95% CI 0.70–0.94, *I*^2^ = 67%). NRFS to RFS total prevalence was 10% (95% CI 0.06–0.15, *I*^2^ = 83%; Fig. 5), while total prevalence of RFS to NRFS was 2% (95% CI 0.01–0.03).

### Foot Strike Pattern and Performance

Of the studies that made an assessment of the relationship between foot strike pattern and performance, 5 found there to be a quantifiable difference in favour of the NRFS pattern being faster compared to RFS [1, 2, 20, 24, 26], 1 study reported a performance benefit to NRFS in women but not men [14], 4 studies reported no benefit of either NRFS or RFS [3, 21–23], and no studies reported a performance benefit of RFS over NRFS (Table 1).

### Asymmetry

Seven of the 12 included studies attempted to quantify and record asymmetry in foot strike pattern [3, 19–22, 24, 26]. These values ranged from as low as 0.7% prevalence in a recreational marathon [20], to as high as 25.9% in an event of the same distance and standard of athlete [24] (Table 1).

## Discussion

This is the first review to assess and quantify the literature base pertaining to foot strike pattern prevalence, foot strike pattern change with increased distance and assessment of the interaction between foot strike pattern and performance within the context of overground long-distance running. The vast majority of distance runners consistently run with an RFS pattern, the prevalence of which is seen to increase with distance. Furthermore, a proportion of runners appear to change foot strike pattern as distance increases and this pattern of change occurs almost exclusively in one direction (NRFS to RFS). Furthermore, inconclusive evidence exists of a performance advantage being associated with the NRFS pattern over the RFS pattern.

Across all studies, 79% (95% CI 0.70–0.86) of runners were observed to use an RFS strike pattern early in a run (Fig. 2) and with increased distance, this prevalence became more pronounced, reaching a value of 86% (95% CI 0.85–0.88; Fig. 2). Foot strike pattern was first captured at different distances from the starting point, ranging from as early as 3 km [1, 25] to as far as 16.5 km [23] into the run. This was also true for the final checkpoint distance in studies that included more than one check point, ranging from 7.78 km [19] to 90.3 km [23]. Disparities in foot strike capture location between studies demonstrate that there is no accepted standard in this particular field of research. However, it should be noted that despite this, all studies except 3 [2, 23, 24] placed their initial (or only) foot strike capture checkpoint at the 10 km mark or earlier. Of note, 2 of the included studies were conducted in trail running settings [22, 23]; a different terrain compared to road surface that has the potential to alter foot strike pattern and biomechanics [27, 28]. Furthermore, 3 studies were conducted on entirely elite running cohorts [2, 14, 19], a population with a greater tendency to use non-rearfoot striking patterns compared to recreational runners [14]. These varying factors inherent within the cohort of studies included likely led to the high heterogeneity observed (Fig. 2: *I*^2^ = 98%); furthermore, the relatively small pool of literature meant that sub-analyses were not possible. While individual studies have previously attempted to quantify the proportion of athletes that run with each of the main categories of foot strike pattern [1–3, 14, 19–26], this review is the first to collate findings from the literature base for overground distance running and provides prevalence data representative of the global literature. As such, researchers, coaches and athletes along with key stakeholders such as shoe manufacturing companies can have greater confidence about the prevalence of foot strike patterns in their respective work.

The prevalence of runners who changed foot strike pattern between the first and last checkpoints was observed to be 11% (95% CI 0.07–0.16). Six of the included studies were designed in a fashion to enable this analysis, each containing differences in total number of participants, total race distance and standard of athlete, which are all factors that might help to explain the heterogeneity observed between studies. Participant sample size in particular appears to be important when considering this phenomenon, with the observation approximating more consistent values when this is factored into the analysis. Of the 6 studies, 3 contained similar participant sample sizes of 286 [3], 316 [23] and 459 [1] and provided similar prevalence estimates relating to total change in foot strike with 10%, 7% and 15%, respectively (Fig. 4). It has been postulated that highly trained athletes could be less prone to foot strike pattern change due to fatigue resistance in the plantar flexor muscle complex of the lower limb [1, 14, 29]. This notion is both supported and challenged by the results of two studies using elite running cohorts, with 0% foot strike pattern change observed across a 10 km race [19] and 20% in a marathon [14]. These incongruent results are potentially explained by the larger total race distance and increased demand of the muscle tendon units during a marathon event when compared to the shorter race [19]. However, with only 12 [19] and 149 [14] total participants included in these analyses, it is also possible that such observed results might simply be an artefact of the smaller sample sizes contained within these studies. A similar argument could be placed for the study that displayed a 4% prevalence in total change of foot strike pattern [25], with this outlier containing only 23 participants in total. Due to the relatively infrequent prevalence of runners who are prone to changing foot strike with increased distance, a large enough sample appears to be requisite in order for this observation to surface reliably within data sets.

Foot strike pattern change with increased distance appears to usually occur in one direction, with 84% of runners who changed foot strike pattern doing so from NRFS to RFS, a phenomenon observed to be five times more prevalent than the converse. This observation seems apparent over multiple distance settings, including the marathon [3, 14] and shorter format distance racing [1]. An accepted mechanism to explain this observation is yet to be clarified in the literature. Possible explanations offered by authors currently revolve around the potential impacts that fatigue, running speed and experience have on foot strike patterns as running distance increases [1, 3, 14]. While the mechanism appears unclear, this review has now established the observation to be consistent in that the pattern of foot strike change, when it does occur, is reported most often in the same direction.

The relationship between foot strike pattern and running performance displayed inconclusive evidence in support of the NRFS pattern conferring a competitive advantage over the RFS pattern. Discussion around the potential improvements in performance garnered by using a non-rearfoot striking pattern has served as topics of debate in previously published literature [1, 4, 5, 7, 14, 15]. Individual studies have observed that top finishers of distance running events tend to use an NRFS pattern [1, 2, 13], while others have not been able to replicate the observation both in recreational [3] and elite [14, 15] running cohorts. Up until this point, no review of the literature pertaining to the interplay between foot strike patterns and performance in the overground distance running setting has been available. Papers reviewed in this analysis employed various methods of assessment in an attempt to quantify and report on the interaction, making comparison difficult on raw data alone. In an attempt to combine the results of these studies, a binary transformation was applied to the pre-existing data, reducing the findings of individual studies to either display RFS or NRFS patterns as being faster, or not as previously described. When quantified and applied to all standards of athlete, there appears to be an inconclusive bias in results towards the NRFS pattern being associated with a performance benefit over the RFS pattern.

Finally, asymmetry of foot strike pattern (difference between left and right feet) was seen to display inconsistent results. Prevalence of the asymmetrical running foot strike pattern within this cohort of studies ranged from 0.7% [20] to 25.9% [24], with the remaining studies displaying values falling between these two extremes [3, 19, 21, 22, 26] (Table 1). Such high variability observed between studies suggests potential disparities exist regarding the categorisation and reporting of asymmetrical foot strike patterns. As such, it is presently difficult to consolidate this aspect of the literature and further research is required to more confidently account for asymmetry prevalence.

## Conclusion

The vast majority of distance runners use a rearfoot strike pattern and the proportion of runners who employ this pattern rises as distance increases. A proportion of runners display a change in foot strike pattern with increased distance, with this phenomenon occurring almost entirely from non-rearfoot strike to rearfoot strike. Finally, there appears to be inconclusive evidence to support a performance benefit associated with non-rearfoot striking over rearfoot striking. The inclusion of both recreational and elite cohorts, across multiple distances and terrains, allows the current findings of this review to be applied to a broad population of runners.

## Supplementary Information

**Additional file 1**. PRISMA Checklist.

## Data Availability

Data can be made available from the corresponding author on request.

## Abbreviations

FFS: Forefoot strike
MFS: Midfoot strike
NRFS: Non-rearfoot strike
PRISMA: Preferred Reporting Items for Systematic Reviews and Meta-Analyses
RFS: Rearfoot strike
